# Efficient and error-free correction of sickle mutation in human erythroid cells using prime editor-2

**DOI:** 10.3389/fgeed.2022.1085111

**Published:** 2022-12-20

**Authors:** Anila George, Nithin Sam Ravi, Kirti Prasad, Lokesh Panigrahi, Sanya Koikkara, Vignesh Rajendiran, Nivedhitha Devaraju, Joshua Paul, Aswin Anand Pai, Yukio Nakamura, Ryo Kurita, Poonkuzhali Balasubramanian, Saravanabhavan Thangavel, Srujan Marepally, Shaji R. Velayudhan, Alok Srivastava, Kumarasamypet M. Mohankumar

**Affiliations:** ^1^ Centre for Stem Cell Research (a Unit of inStem, Bengaluru), Christian Medical College Campus, Vellore, India; ^2^ Sree Chitra Tirunal Institute for Medical Sciences and Technology, Thiruvananthapuram, India; ^3^ Manipal Academy of Higher Education, Manipal, Karnataka, India; ^4^ Department of Haematology, Christian Medical College and Hospital, Vellore, India; ^5^ Cell Engineering Division, RIKEN BioResource Center, Ibaraki, Japan; ^6^ Research and Development Department, Central Blood Institute Blood Service Headquarters, Japanese Red Cross Society, Tokyo, Japan

**Keywords:** prime editing, sickle cell anaemia, CRISPR, haemoglobinopathies, beta-globin

## Abstract

Sickle cell anaemia (SCA) is one of the common autosomal recessive monogenic disorders, caused by a transverse point mutation (GAG > GTG) at the sixth codon of the beta-globin gene, which results in haemolytic anaemia due to the fragile RBCs. Recent progress in genome editing has gained attention for the therapeutic cure for SCA. Direct correction of SCA mutation by homology-directed repair relies on a double-strand break (DSB) at the target site and carries the risk of generating beta-thalassaemic mutations if the editing is not error-free. On the other hand, base editors cannot correct the pathogenic SCA mutation resulting from A > T base transversion. Prime editor (PE), the recently described CRISPR/Cas 9 based gene editing tool that enables precise gene manipulations without DSB and unintended nucleotide changes, is a viable approach for the treatment of SCA. However, the major limitation with the use of prime editing is the lower efficiency especially in human erythroid cell lines and primary cells. To overcome these limitations, we developed a modular lenti-viral based prime editor system and demonstrated its use for the precise modelling of SCA mutation and its subsequent correction in human erythroid cell lines. We achieved highly efficient installation of SCA mutation (up to 72%) and its subsequent correction in human erythroid cells. For the first time, we demonstrated the functional restoration of adult haemoglobin without any unintended nucleotide changes or indel formations using the PE2 system. We also validated that the off-target effects mediated by the PE2 system is very minimal even with very efficient on-target conversion, making it a safe therapeutic option. Taken together, the modular lenti-viral prime editor system developed in this study not only expands the range of cell lines targetable by prime editor but also improves the efficiency considerably, enabling the use of prime editor for myriad molecular, genetic, and translational studies.

## Introduction

Sickle cell Anaemia (SCA) is one of the most prevalent monogenic disorders worldwide, caused by a single transverse point mutation (GAG>GTG) in the sixth codon of the beta-globin (*HBB*) gene (Brittenham et al., 1985). This mutation results in the replacement of a polar amino acid residue (glutamate) with a non-polar residue (valine) causing adult haemoglobin to polymerise under conditions of low oxygen saturation and RBCs acquire the characteristic sickle shape with increased susceptibility to haemolysis. SCA patients present with haemolytic anaemia as well as other acute and chronic complications, such as recurrent episodes of pain and organ damage due to the obstruction of blood vessels by the distorted RBCs (Kavanagh et al., 2022). Being an autosomal recessive disorder, HbS mutation can be present in heterozygous (Sickle Cell Trait), homozygous (Sickle cell Anaemia), or compound heterozygous state (in combination with other structural or functional beta-globin variants) and this spectrum of diseases is commonly referred to as Sickle Cell Disease (SCD). Sickle Cell trait is a less adverse condition and patients do not present with clinical complications unless exposed to extreme conditions that can induce sickling of RBCs but SCA is a severe condition with reduced life expectancy and vast healthcare burden (Kato et al., 2018). The treatment approaches currently in practice are the administration of hydroxyurea that can elevate non-sickling fetal haemoglobin or blood transfusions with iron chelating agents that can improve the number of non-sickling RBCs(Platt et al., 1984; Sheth et al., 2013). The only curative approach approved for SCA is allogeneic stem cell transplant but is limited by the lack of availability of HLA matched donors and the risk of graft rejection which has prompted the research for other curative approaches (Salinas Cisneros and Thein, 2020).

Gene therapy and *ex vivo* genome editing approaches have proven successful in preclinical studies and are now being tested in ongoing clinical trials (Morgan et al., 2017; Magrin et al., 2019). Lenti-viral based approaches to complement the defective adult haemoglobin with non-sickling fetal or adult haemoglobin are currently the preferred strategies, but the long-term safety issues due to insertional mutagenesis are unknown (Pawliuk et al., 2001; Ribeil et al., 2017; Kanter et al., 2022). CRISPR/Cas based approaches are now gaining recognition as *ex vivo* gene editing tools owing to the efficiency and versatility (Barbarani et al., 2020; Prasad et al., 2021). Previous studies have attempted the correction of HbS mutation using Homology Directed Repair (HDR) with Cas9 but suffered from low efficiency and engraftment potential (DeWitt et al., 2016). Furthermore, unrepaired indels from DSB at the target site would result in a more severe beta-thalassemia phenotype rather than correcting SCD. Over the years attempts at improving the HDR efficiency and reducing indel formation at the target site have been successful but still do not overcome the general risks posed by DSB such as large deletions, translocations, chromothripsis and p53 activation (Leibowitz et al., 2021; Papathanasiou et al., 2021; Amendola et al., 2022).

Base editing for the substitution of key nucleotides at the gamma-globin regulatory elements to mimic the naturally occurring HPFH conditions thereby elevating fetal haemoglobin is a potential alternative strategy for the treatment of SCD (Psatha et al., 2018, 11; Esrick et al., 2021, 11; Ravi et al., 2022). The use of single-strand nick at the target site keeps the generation of indels to a minimum and base editors were shown to be highly efficient in installing transition mutations (by deaminating the purine or pyrimidine to the respective bases) in CD34+ HSPCs without affecting the engraftment potential (Zeng et al., 2020). However, correction of transverse point mutation (T>A) is not possible with base editors described so far which poses a challenge in the treatment of SCD (Anzalone et al., 2020). Recently, the conversion of sickle beta-globin to a non-sickling haemoglobin variant HbG Makassar, using adenosine base editor (GTG>GCG, Val > Lys) has proven to be successful *in vitro* and in SCD mouse models. However, this approach uses a more common NRCH PAM which increases the number of potential off-target sites. Further, the functional utility of this approach needs to be extensively characterised for therapeutic applications (Newby et al., 2021).

Prime editing is a CRISPR-based novel gene editing tool that allows the installation of precise mutations in the target locus without causing double-strand break (DSB) and limited PAM constraints. PE relies on a reverse transcriptase fused to a Cas9 nickase to incorporate the mutations encoded by the prime editing guide RNA (pegRNA) into the target DNA (Anzalone et al., 2019). This approach offers several advantages over conventional DSB based approaches as well as base editing in therapeutic gene editing. PE enables precise introduction of point mutations including transition mutations, insertions, or deletions in the genomic region of interest while overcoming the limitations associated with DSB, thus expanding the scope of gene therapy (Anzalone et al., 2020). Prime editing has been successfully harnessed in proof-of-concept studies for correcting genetic disorders as well as developing cellular and animal models for genetic disorders (Schene et al., 2020; Petri et al., 2021; Doman et al., 2022; Lu et al., 2022). However, the efficiency of prime editing is low compared to other approaches and the cells that can be edited are limited. One approach to increase the editing efficiency was the PE3 system that uses an additional gRNA that nicks the unedited strand in the PE3 system along with the targeting pegRNA and prime editor (PE2) but was reported to generate a higher proportion of InDels. Till date prime editing has not been demonstrated for the correction of HbS mutation in human erythroid cells and the functional evaluation and safety profiling have not been carried out, which limits its clinical application although Anzalone et al. had described the proof of concept for correction of SCA using HEK293T cells.

In this study, we describe the use of prime editing for the correction of HbS mutation in human erythroid cell line (HUDEP2) and demonstrate the reconstitution of functional adult haemoglobin in the SCA model with high efficiency and precision. As the expression of PE components for a longer duration of time might increase the editing efficiency, we developed a selectable dual lenti-viral system for delivery of PE and pegRNA that showed highly efficient editing than previously reported approaches in human erythroid and other cell lines. We hope this study would pave the foundation for therapeutic *ex vivo* gene editing of HSPCs for the treatment of SCD and other beta-haemoglobinopathies. Further, the modular lenti-viral PE delivery system that we developed would expand the type of cells targetable by prime editing, enabling efficient screening and functional evaluation of prime editing for various genetic diseases.

## Materials and methods

### Plasmids constructs

Plasmids pCMV-PE2 (Addgene #132775), pU6-pegRNA-GG-acceptor (Addgene #132777) and pU6-Sp-pegRNA-HEK3_CTT_ins (Addgene #132778) were a gift from David Liu. pMD2.G and psPAX2 (second-generation lenti-viral packaging construct, Addgene #12259, #12260) were a gift from Didier Trono. pLenti_PE2_P2A_Puro vector was constructed by assembling amplified PE2 (from Addgene #132775; primers listed in Table) into BamH1 and Nhe1 digested pLenti-ABERA-P2A-Puro vector (Addgene#112675; a gift from Lucas Dow) using NEBuilder HiFi DNA Assembly Master Mix as per the manufacturer’s instructions. Lenti_pegRNA_GFP/RFP vector was constructed by removing the gRNA scaffold from pLKO5.sgRNA.EFS.GFP/RFP (Addgene #57822/57823; Gift from Benjamin Ebert).

### Design and cloning of pegRNA

PegRNA sequences were selected from previous publication (Details in Supplementary Table). pU6-Sp-pegRNA-HEK3_CTT_ins was obtained from Addgene (#132778); all other pegRNAs used in the study were cloned in Lenti_pegRNA_GFP/RFP vector. Spacer, extension template and scaffold sequences were obtained as oligonucleotides with respective overhangs in the top and bottom strands to facilitate Goldengate assembly (Supplementary Figure S1A). Oligo annealing was carried out for respective sets as per the previously published protocol (Ravi et al., 2022). Briefly, 1 µL each of forward and reverse oligonucleotides (100p.m.), 0.5 µL of T4 PNK(NEB), 1 µL of T4 DNA ligase buffer (NEB) and 6.5 µL of H_2_O was mixed and oligo annealed. After annealing the top and bottom strands of the respective components, golden gate assembly was carried out using Bsmb1 V2(NEB) digested Lenti_pegRNA_GFP/RFP vector as the backbone. In brief, 1 µL each of 1:10 diluted annealed oligos (total 3 µL), 50 ng of digested vector, 0.5 µL of T4 DNA ligase, 0.25 µL of BsmB1 V2 enzyme and 1 µL of T4 DNA ligase was mixed and volume was made up to 10 µL using nuclease-free water. The golden gate assembly reaction was carried out as follows: (42°C, 3 min → 16°C, 3 min) x 30 cycles, 60°C, 5 min → 4°C. The reaction product was then transformed into DH10B competent cells and plated in LB agar containing 100 μg/ml of ampicillin for selection. The selected colonies were verified using colony PCR followed by sanger sequencing as per published protocol (Ravi et al., 2022). The positive clones were inoculated further in LB broth and cultured overnight. Plasmids were isolated using NucleoBond Xtra Midi EF (Macherey-Nagel) kit according to the manufacturer’s instruction.

### Cell culture

HEK293T cells were cultured in DMEM (HyClone) supplemented with 10% FBS and 1x Penicillin Streptomycin. K562 cells were maintained in RPMI supplemented with 10% FBS and 1x Penicillin Streptomycin HUDEP2 cells were cultured in StemSpan™ SFEM II (STEMCELL Technologies) supplemented with 50 ng/ml SCF (ImmunoTools), 3 U/ml EPO (Zyrop 4000 IU Injection), 1x Pen-Strep, 1 µM dexamethasone (Alfa Aesar), 1 μg/ml doxycycline (Sigma-Aldrich) and 1x l-Glutamine 200 mM (Gibco™) (Kurita et al., 2013). Erythroid differentiation of HUDEP2 cells was carried out using two-phase erythroid differentiation media. Phase I media consisted of IMDM glutamax (Gibco), 3% AB serum (MP Biomedicals), 2% FBS, 0.1% insulin solution human (Sigma-Aldrich), 3 U/ml Heparin sodium salt (MP Biomedicals), 200 μg/ml Holo Transferrin (BBI Solutions), 3 U/ml EPO, 10 μg/ml SCF, 1 ng/ml IL3 (Immuno Tools), 1x Pen-Strep, and 1 μg/ml doxycycline. Phase II media is similar in composition except that it is devoid of doxycycline and contains 500 μg/ml of holotransferrin. Around one million cells were used to set up differentiation. Media change was done on day-3 and cells were shifted to phase II media on day-6. The culture was terminated on day-9.

### Transfection

2 × 10^5 HEK293T cells were seeded in a 12-well cell culture dish. Upon reaching 80% confluency, the cells were transfected with the appropriate plasmids using FuGENE-HD (Promega) as per the manufacturer’s protocol. Briefly, 100 µL OptiMem, 1,000 ng of plasmids and 3 µL of FuGENE-HD (1:3 ratio; scaled up when required) was mixed well, incubated for 15 min, and added to the cells. Media was changed 24 h later and cells were dissociated and taken for analysis from 72 h s onwards. Prime Editor to pegRNA ratio was maintained at 3:1.

### Electroporation

pegRNA plasmid was electroporated into K562/HUDEP2 PE stables using Lonza 4D electroporator using the program EN138. 2 µg of plasmid was nucleofected to one million cells as per the manufacturer’s protocol.

### Lenti-virus production and transduction

About 2 × 10^6 HEK293T cells were seeded in a 10 cm cell culture dish with DMEM (HyClone) containing 10% FBS and 1x Pen-Strep. At 80% confluency, 2.5 μg of pMD2.G, 3.5 μg of psPAX2 and 4 μg of lenti-viral vector (PE2 or pegRNA constructs) were transfected using FuGENE-HD as per the manufacturer’s protocol. Viral supernatant was then collected after 48 h and concentrated using Lenti-X Concentrator (Takara). The concentrated pellet was resuspended in 500 μL 1xPBS and stored at −80°c until use. For transduction, 0.2 million cells were taken in 12 well plate with 10 µL HEPES 1M buffer (Gibco) and 1 µL of polybrene (Sigma Aldrich; 6 mg/ml concentration). Virus was thawed, 100 µL added to each well and spinfected at 800 g for 30 min at room temperature. After 48 h s media was changed and 1 μg/ml puromycin (Gibco) was added for stable preparation. The transduction efficiency of pegRNA was analysed using GFP/RFP expression by flow cytometry (BD celesta or BC Cytoflex).

### DNA isolation, sequencing, and analysis of editing efficiency

To determine the prime editing efficiency, genomic DNA from the edited cells was isolated using DNA isolation kit (Qiagen). For experiments where editing was tested sequentially over time, DNA was isolated from about 0.2 million cells using quick DNA extract (epicentre). The target region was amplified using the respective primers (Supplementary Tables), and the amplified product was Sanger sequenced. The sequencing data were analysed either using Synthego ice (for insertions/deletions) (Conant et al., 2022)or EditR (for substitutions) (Kluesner et al., 2018). For next-generation sequencing, primer sets with ∼250 bp amplicon size and appropriate adapters were used (Listed in Table). NGS data was analysed using Crispresso-2 (Clement et al., 2019).

### cDNA sequencing

For checking the edits at RNA level, total RNA from the cells was isolated using NucleoSpin RNA kit (Macherey-Nagel) and reverse transcribed to cDNA using iScript cDNA synthesis kit (BioRad) as per the manufacturer’s protocol. cDNA was PCR amplified using the primers listed in Table and Sanger sequenced. Editing efficiency was analysed using EditR.

### MLPA

MLPA was performed using SALSA MLPA probe mix P102 HBB with 100 ng/μL DNA as per the manufacturer’s protocol. The data was analysed and visualised using Coffalyser software.

### HPLC

HPLC was used to quantify haemoglobin variants and individual globin chains in differentiated HUDEP2 cells. Cells were collected, washed with 1x PBS, and resuspended in double distilled water. The cells were then subjected to sonication following which the suspension was centrifuged at 14,000 rpm for 10 min at 4°C. The supernatant was collected and stored at -80 until analysis. The samples were analysed using VARIANT II Haemoglobin Testing System (Bio-Rad) for haemoglobin variants and data was quantified by the Bio-Rad’s Clinical Data Management (CDMTM) Software. Individual globin chains were quantified using Reverse phase HPLC (Shimadzu Corporation-Phenomenex).

### VCN analysis by real-time PCR

Vector copy number was analysed in genomic DNA isolated from the stable cell lines. Primers targeting the Cas9 gene were used to detect the integration of prime editor. A primer set targeting Factor IX gene was used as an internal reference. pCMV-PE2 (Addgene #132775) and an in-house plasmid construct with Factor IX CDS were used as standards.

### Prime editor expression analysis by real-time PCR

RNA was isolated from the cells using NucleoSpin RNA kit (Macherey-Nagel) and reverse transcribed to cDNA using iScript cDNA synthesis kit (BioRad). The relative expression of prime editor in the stables compared to the respective wild-type cells was quantified using primers targeting Cas9. GAPDH was used as the normalising internal control. The PCR reaction was carried out using SsoFast EvaGreen Supermixes (Bio-Rad) in QuantStudio six Flex Real-Time PCR System (Applied Biosystems) after three fold dilution of cDNA samples as reported previously (Ravi et al., 2022).

### Single cell sorting

Single-cell sorting to obtain isogenic clones were done using BD FACS Aria cell sorter in 96-well format. After Sanger sequencing, desired clones were expanded and used for further analysis.

### HbF intracellular staining

To evaluate the frequency of HbF-positive cells, the cells were fixed, permeabilized, and intracellular staining was performed using Fetal Haemoglobin Monoclonal Antibody (HBF-1), APC (Invitrogen) as previously described (Canver et al., 2015). The stained cells were analysed by Flow cytometry (CytoFLEX LX Flow Cytometer—BC) to measure the number of HbF-positive cells.

### Off-target analysis

Cas-OFFinder/Cosmid was used to predict the gRNA-dependent off-target regions, up to three mismatches were allowed in selecting targets. The top four targets were amplified and sequenced using Illumina MiSeq platform (Supplementary Table). CRISPResso2 was used for data analysis and visualisation.

### Statistics

All analyses were performed using GraphPad Prism V8.1 and *p*-value < 0.05 was considered statistically significant.

## Results

Prime editing has tremendously expanded the scope of therapeutic genome editing with its ability to correct various disease-causing mutations resulting from substitutions, small insertions, and deletions (Indels) without generating undesired editing outcomes. One of the major limitations in taking this approach forward for clinical translation is that the efficiency of prime editing decreases as we move from workhorse cell lines to more specialised cell lines and even further when taken to primary cells (Jin et al., 2021). This decline in efficiency also hinders the appropriate evaluation of on-and off-target effects mediated by the prime editor. Therefore prior to evaluating prime editing in erythroid cell lines for correction of SCA mutation, we aimed at developing a selectable lenti-viral PE system that could not only be used with ease in multiple cell lines but also yield better editing efficiency than the conventional delivery approaches.

### Lenti-viral delivery of prime editor enables efficient editing across multiple target sites in HEK293T cells

We sought to develop a lenti-viral delivery system for prime editing that would ensure constitutive availability of components allowing for maximum editing efficiency. The prospect of cloning prime editor in a single lenti-viral vector has been dismissed previously owing to its large size (Gao et al., 2022). Limited number of studies have used lenti-viral delivery of PE and have relied on the split-intein approach which requires simultaneous delivery of two different constructs and has not shown significant elevation in editing efficiency (Anzalone et al., 2019). Nevertheless, with our past success in cloning base editor into a lenti-viral based vector (Ravi et al., 2022), we considered developing a similar prime editing system, with the PE delivered in one vector and pegRNA in another. We limited the study to PE2 system since multiple publications have shown an increase in indels with the use of additional nicking gRNA in the PE3 system.

**FIGURE 1:**
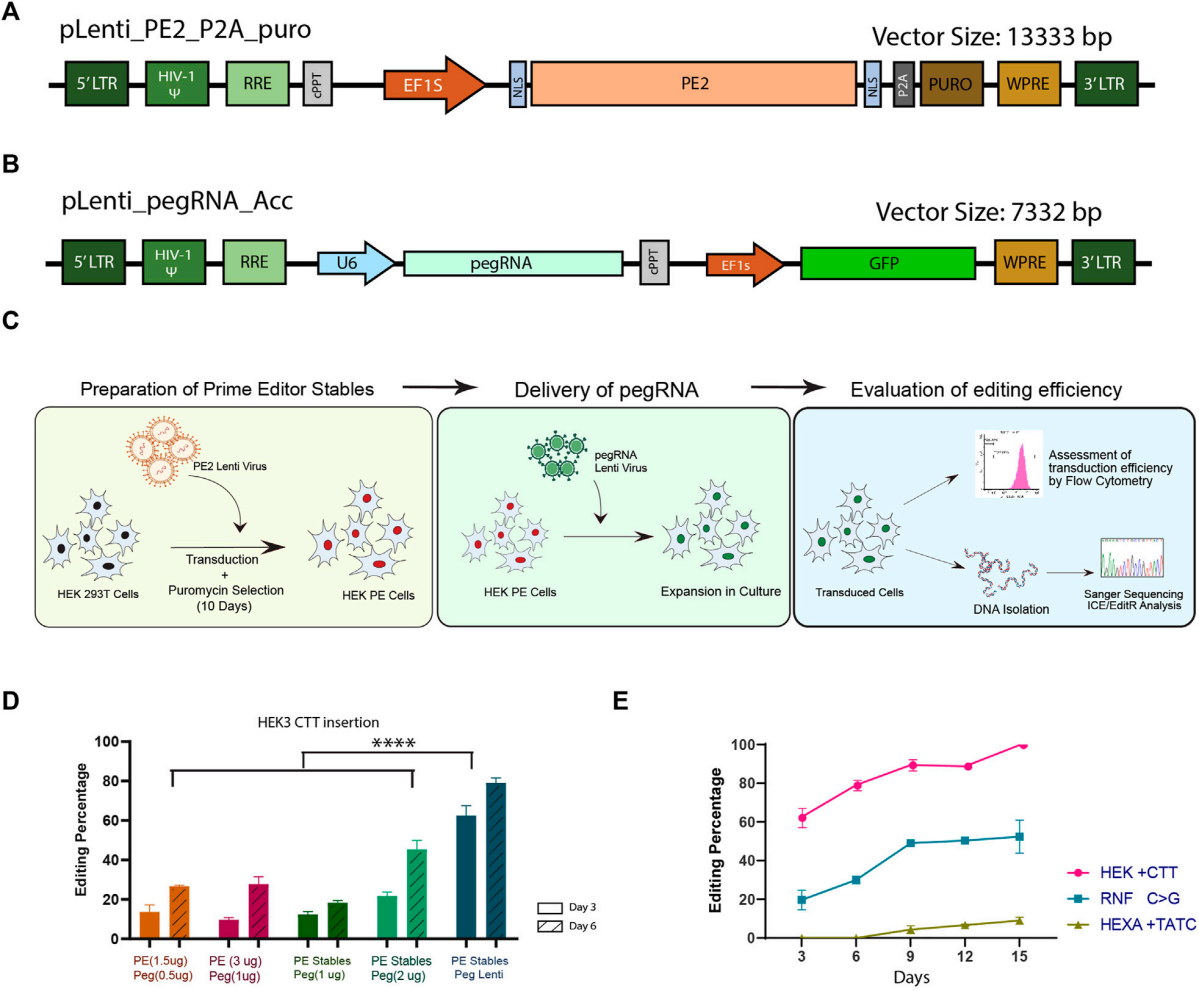
Generation and characterisation of a modular lenti-viral prime editor system in HEK293T cells. Schematic representations of the prime editor **(A)** and pegRNA **(B)** lenti-viral constructs developed in this study. **(C)** Overview of the general workflow for prime editing by the dual lenti-viral system. **(D)** Evaluation of prime editing efficiency for CTT insertion in HEK3 locus; The efficiency of dual lenti-viral delivery of the components (blue) was compared against transfection of pegRNA plasmid in HEK293T PE stables (dark and light green) and dual transfection of PE2 and pegRNA (orange and magenta) by sanger sequencing at two different time points. **(E)** Prime editing efficiency by dual lenti-viral delivery at 3 different genomic loci evaluated by sanger sequencing over a period of 15 days. Results from three replicates are plotted as mean ± SD; Asterisks indicate the level of statistical significance; One way ANOVA with Tukey’s test was used for comparison between the groups. (****p < 0.0001).

Prime editor (PE2) sequence (Anzalone et al., 2019) was cloned into a lenti-viral backbone driven by EF1 alpha (short) promoter which is known to give better transgene expression and stability across various cell lines (Zafra et al., 2018). The PE sequence was linked *via* self-cleaving peptide (P2A) to puromycin resistance gene that would allow the selection of cells with stable expression of prime editor (Figure 1A). Subsequently, the pegRNA was cloned into a lenti-viral backbone driven by U6 promoter which co-express GFP, enabling positive selection and validation of transduction efficiency (Figure 1B; Supplementary Figure S1A). We presumed that the relatively low efficiency of PE compared to the other genome editing strategies could be overcome by prolonged expression of PE through the dual lenti-viral system which will ensure the availability of PE components for multiple rounds of cell division till the desired conversion is achieved at the target site. Further, this system would enable the generation of cell lines stably expressing prime editor, which can be used for simultaneous arrayed screening of multiple pegRNAs overcoming the inconsistencies associated with transfection of large PE plasmid.

We first validated the efficiency of the lenti-viral PE system in HEK293T cells by inserting a CTT sequence in HEK3 locus using previously validated pegRNA (Anzalone et al., 2019). HEK293T PE stables were generated by lenti-viral transduction of PE2 construct followed by puromycin selection for 10 days (Figure 1C). The HEK293T PE stables were either transduced with lenti-virus of pegRNA for inserting the CTT sequence or transiently transfected with pegRNA construct described by Anzalone et al. This approach was then compared against transient transfection of both PE2 and pegRNA in HEK293T cells. Three and six days after delivery of the components, cells were collected, genomic DNA isolated and subjected to sanger sequencing to assess the editing efficiency. We noted a significant elevation in editing efficiency in samples transduced with lenti-virus of pegRNA compared to the pegRNA transfected samples (Figure 1D). This could be accounted for by the perpetual expression of PE components in the transduced samples, as lenti-virus integrates into the host genome. By day-6, HEK293T PE cells transfected with pegRNA also showed a higher editing efficiency than dual transfection of PE2 and pegRNA which might be due to the increase in the delivery dosage of pegRNA alone when compared to the dual transfection. Higher editing efficiency noted with the increase in the concentration of components delivered hints that the availability of components might be a limiting factor in determining the editing efficiency. However, we did not find any discernible difference in the editing efficiency when the concentration of PE2 and pegRNA was increased in the dual transfection, probably because of the limitations in transfecting the large sized PE2 constructs. Though the editing efficiency of pegRNA alone transfected in PE stable might be lower than what would be expected with lenti-viral delivery, transfection at higher doses enables screening of multiple pegRNAs without the need for producing lenti-virus for each target.

We further tested the system by creating mutations at two additional genomic loci with previously described pegRNAs for *RNF* +1C>G conversion and a TATC insertion in *HEXA* locus (Anzalone et al., 2019). Lenti-viral pegRNAs were transduced into HEK293T PE cells and the editing efficiency was evaluated by sanger sequencing over a period of 15 days. Although the transduction efficiency was similar across all three target sites (Supplementary Figure S1B), editing efficiency varied considerably between the targets (Figure 1E). As has been previously observed (Wolff et al., 2021), the pegRNA for insertion of CTT sequence in HEK3 locus resulted in the highest editing efficiency starting with∼ 60% insertion on day-3 and reaching ∼100% by day-15 (Figure 1E). RNF C>G conversion showed modest editing of ∼20% on day-3 and increased up to ∼50% on day-15. On the other hand, HEXA TATC insertion did not show any appreciable editing till day-9 and the maximum efficiency obtained was around 10% on day-15. These data suggest that even though the availability of pegRNA and prime editor is not limited, the editing efficiency and the installation of the desired mutation might vary from one locus to the other, necessitating multiple steps of optimisation at each of the desired loci. Taken together, the modular prime editing approach that we have described is versatile for studies requiring extensive screening and validation of pegRNAs.

### Efficient prime editing in the human erythroid cell lines mediated through lenti-viral delivery system

**FIGURE 2:**
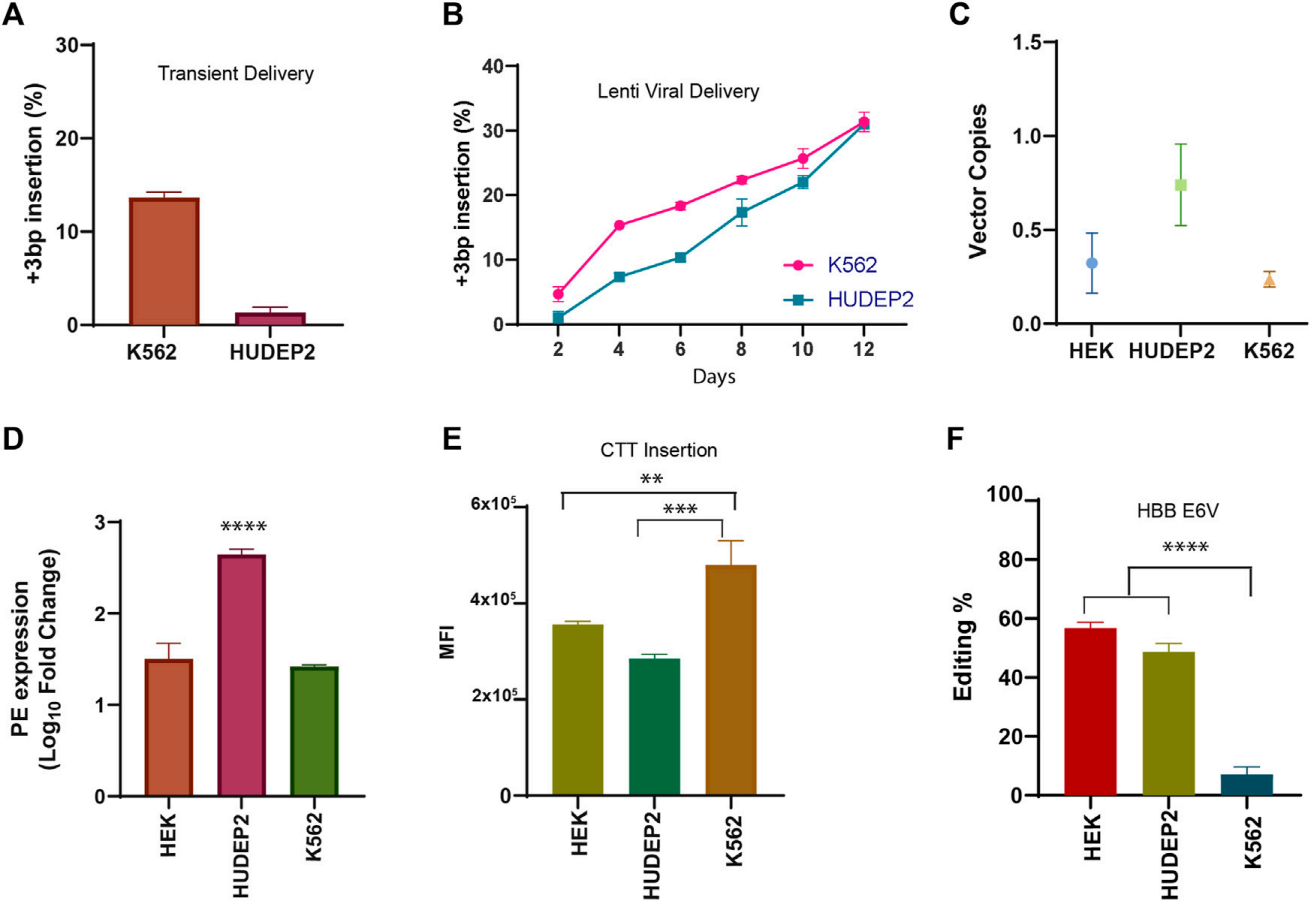
Validation of prime editing in human erythroid cell lines **(A)** Efficiency of CTT insertion in HEK3 locus in K562 and HUDEP2 PE cells upon nucleofection of pegRNA plasmid. **(B)** Dynamics of CTT insertion efficiency in HEK3 locus by lenti-viral delivery in K562 and HUDEP2 cells evaluated by sanger sequencing **(C)** The number of prime editor vector copies integrated in the genome of HEK293T, HUDEP2, and K562 PE cells assessed by RT PCR. **(D)** Expression of prime editor in PE stable cell lines normalised to respective wild-type cells. **(E)** Mean Fluorescence Intensity of GFP expression in the PE stable cells transduced with pegRNA for CTT insertion. **(F)** Efficiency of installation of Sickle Mutation (A>T conversion) in the beta-globin gene in the three different cell lines by lenti-viral delivery measured by sanger sequencing 12 days after transduction. Results from three replicates are plotted as mean ± SD; asterisks indicate the level of statistical significance; One way ANOVA with Tukey’s test was used for comparison between the groups. (**p < 0.01, ***p < 0.001, p****<0.0001).

Encouraged by the efficiency and versatility of the PE lenti system in HEK293T cells, we evaluated the prime editing efficiency in two commonly used human erythroid progenitor cell lines (K562 and HUDEP2) that are widely used as preclinical models in hematological studies. Although prime editing has been tested in K562 cells by different approaches, the editing efficiency was lower compared to HEK293T cells (Wolff et al., 2021) and the prime editing efficiency reported in HUDEP2 cells was very minimal (Zhang et al., 2022). We hypothesised that the lower efficiency in these cell lines could be overcome by a constitutive expression of prime editing components. We first tested whether a transient delivery of pegRNA into K562 and HUDEP2 cell lines stably expressing PE could edit efficiently as was seen in HEK293T PE cells and compared the efficiency against dual lenti-viral delivery in human erythroid cell lines. The efficiency of prime editing in both the erythroid cell lines was validated using the pegRNA for CTT insertion in HEK3 locus, which showed the highest editing in the HEK293T cells. Upon nucleofection of pegRNA construct, we observed moderate editing efficiency (13.6%) in K562 cells and very low frequency of 3bp insertion (1.33%) in HUDEP2 cells by day-3 (Figure 2A; Supplementary Figure S2A). On the other hand, by lenti-viral mediated transduction of pegRNA, we achieved efficient insertion of the CTT sequence in K562 cells which was either comparable to or better than previously described prime editing delivery approaches (Wolff et al., 2021) (Figure 2B). We observed almost comparable editing efficiency for transient (day-3) vs. lenti-viral delivery (day-4) in K562 cells (13.6% vs. 15.33%). However, we noticed an increased editing efficiency by lenti-viral mediated delivery when compared to transient transfection in HUDEP2 cells (1.33% vs 7.33%). The editing efficiency in K562 cells was higher than HUDEP2 cells in the initial days but over time reached similar efficiency. The maximum efficiency that could be achieved by day-12 was around 30% in both cell lines, although the overall efficiency was lower than what was observed in HEK293T cells (∼30% vs. 89%) (Figure 2A; Figure 1E). Surprisingly, we also observed indels in both the cell lines with transient delivery which was not seen with lenti-viral delivery possibly because of the continued availability of pegRNA in the lenti PE2 system for editing until error-free conversion is achieved at the target site (Supplementary Figure S2B).

As all 3 cell lines were shown to have been transduced with both prime editor as well as pegRNA constructs (evidenced by survival in puromycin selection and GFP expression) (Supplementary Figure S2C), we compared whether there is any difference in expression of PE or pegRNA between these cell lines which results in varying editing efficiency at the same target site. The number of PE2 vector copies integrated into the genome for each of the stable cell lines was quantified by RT-PCR. There was no appreciable difference between HEK293T and K562 cell lines while HUDEP2 cells showed a higher number of vector copy integration (Figure 2C). This pattern was also reflected in the expression levels of PE2 mRNA in all these cell lines (Figure 2D). These results from integration and expression pattern of PE2 components did not correlate with the editing efficiency which was higher in HEK293T cells suggesting that prime editor availability might not be the only deciding factor for varying editing outcomes. The mean fluorescence intensity of GFP expression from the pegRNA was also compared in all 3 cell lines and the patterns did not corroborate the editing in the respective cell line (Figure 2E). The difference in editing efficiency might be due to cell-intrinsic factors affecting the editing outcome such as the chromatin accessibility or the presence of mismatch repair proteins (Chen et al., 2021; Ferreira da Silva et al., 2022).

Upon ensuring that the prime editor system works efficiently in both the erythroid cell lines, we went ahead with creation of the HbS mutation (*HBB* E6V) in K562 and HUDEP2 cell lines along with HEK293T cells. We selected the pegRNA that was shown to produce the highest editing efficiency for *HBB* E6V installation in HEK293T cells by Anzalone et al. K562, HUDEP2 and HEK293T cell lines stably expressing PE2 were transduced with pegRNA and were cultured for 12 days before editing efficiency was evaluated by sanger sequencing. Although HEK293T cells showed the highest editing efficiency for HbS creation, the difference was not as marked as observed for CTT insertion in HEK3 locus (Figure 2F). Interestingly, we observed a higher editing frequency for the installation of HbS mutation compared to CTT insertion in HUDEP2 cells suggesting that the prime editing efficiency is dependent on the locus and varies from cell to cell. To our surprise, we did not observe any significant editing in K562 cells although the transduction efficiency was 100% (Supplementary Figure S2D). The result was similar to what was observed by Wolff et al. on day-10 of prime editing for the creation of HbS mutation in K562 cells by piggy-bac transposons. The editing efficiency in these cells might increase over time by prolonging the culture of cells (Wolff et al., 2021). Further experiments need to be performed to evaluate if this minimal editing is in any way associated with the gene expression pattern that varies between K562 and HUDEP2 cells or other epigenetic factors intrinsic to the cell line. In summary, we show efficient prime editing without any indels or bystander edits using lenti-viral system in two different human erythroid cell lines. Lenti-viral mediated delivery of prime editor enables the installation of mutations in cell lines that are not easily transfected, expanding the range of cell lines that can be prime edited. Therefore, with the expanded repertoire of targetable cells, it would be possible to utilise prime editing with its full potential in addressing a wide range of research questions in varied research areas.

### Modelling of sickle cell anaemia in human erythroid cell line by prime editor yields a higher proportion of biallelic HbS mutation

**FIGURE 3:**
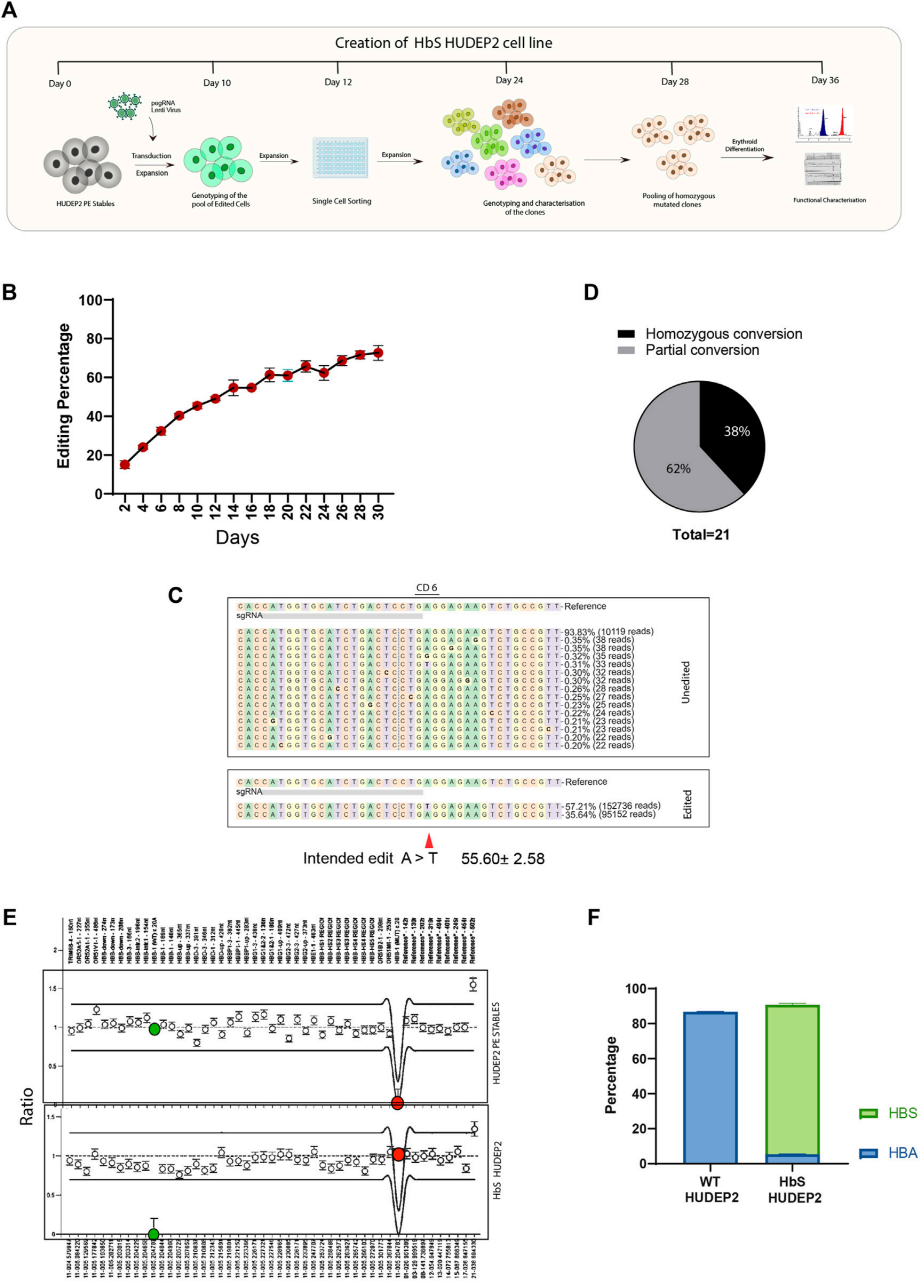
Modelling of Sickle Cell Mutation in human erythroid cells using prime editor **(A)** Schematic representation of the methodology for the creation of sickle mutant cell line using prime editor **(B)** Change in prime editing efficiency for 30 days in HUDEP2 PE cells transduced with pegRNA for installation of HBB E6V mutation measured by sanger sequencing. **(C)** Representative NGS data of overall editing efficiency on the day of single cell sorting **(D)** pie chart showing the proportion of the single cell clones obtained classified according to the genotype at the beta-globin gene **(E)** MLPA analysis chart with the WT probe (Green) and Sickle Mutant probe (Red) highlighted. WT probe amplified exclusively in the unedited cells while the HbS HUDEP2 cells showed amplification with the Sickle probe. **(F)** Percentage of haemoglobin variants after erythroid differentiation of HUDEP2 cells assessed by HPLC. Results from three replicates are plotted as mean ± SD.

Although other groups have previously demonstrated the modelling of SCA in HUDEP2 cells using CRISPR/Cas-9 mediated HDR (Demirci et al., 2020), we evaluated the efficiency of PE utilising the lenti-viral delivery approach to generate a cellular model of SCA in HUDEP2 cells. (Figure 3A). We selected HUDEP2 cells for modelling and correction of HbS mutation as it mimics the adult human erythroid cells and predominantly express beta-globin. We first characterised the dynamics of prime editing for HbS mutation creation by sanger sequencing over a period of 30 days in HUDEP2 PE stables as the PE components were transduced by the selectable lenti-viral vector shown to express for a longer duration of time. Most of the cells were completely transduced with pegRNA as seen by the GFP expression (Supplementary Figure S3A). On day-2 after transduction, the editing efficiency for installing HbS mutation was around 16%, reaching a maximum of ∼70% by day-30 (Figure 3B). There was a consistent increase in editing efficiency during the experimental period which plateaued around day-26. We also evaluated the formation of indels at the nick site and as expected with PE2 system there was no indel formation even at day-30 when the overall editing efficiency had reached 73% (Supplementary Figure S3B). These data provide confidence that prime editor could be used for precise modelling of SCA with the possibility to obtain a greater number of individual clones with the desired mutation compared to Cas9/HDR based approaches.

SCA phenotype is evident when expressed in the homozygous condition because it is inherited in an autosomal recessive manner. Therefore, we performed the single-cell sorting of edited cells on day-12 to obtain homozygous clones of HbS mutation for further phenotypic characterisation. Almost half of the cells were edited on the day of sorting with an overall editing efficiency of ∼55% and no trace of undesired mutations or indels (Figure 3C). Of the 21 clones that proliferated, 38% were homozygous for HbS mutation (8 clones), and the average editing efficiency was 72% (Figure 3D, Supplementary Figure S3C). Further, all but one of the sequenced clones showed at least 50% desired mutation suggesting that all the cells in the pool of edited cells were harbouring the SCA mutation and given more time these cells also might achieve homozygous conversion. We selected the eight clones that showed homozygous conversion of HbS for further experiments. The clones were evaluated for fetal haemoglobin levels to eliminate any variation due to clonality which can interfere with the precise evaluation of sickle or adult haemoglobin. We found six out of eight clones with elevated HbF levels (Supplementary Figure S3D). We pooled the clones that showed lower HbF and expanded further before proceeding with functional characterisation.

Even after multiple passages, the homozygosity of the edited cells was maintained without any undesired edits or indel formation (Supplementary Figure S3E). We performed Multiplexed Ligation Dependent Probe Amplification (MLPA) in the beta-globin locus using the genomic DNA and reconfirmed the installation of HbS mutation, absence of normal genotype (HBB E6), and the intactness of the other regions in the globin locus (Figure 3E). We further differentiated the clones in the erythroid medium for 8 days and the haemoglobin variants produced were analysed by HPLC. The pool of homozygous clones showed almost complete presence of sickle haemoglobin in contrast to the unedited control (Figure 3F). There was no significant alteration in either HbF or HbD which suggests the successful creation of sickle cell mutation in HUDEP2 cells that mimic the human disease both phenotypically as well as genotypically. This pool of homozygous clones (Further referred to as HbS HUDEP2 Cells) was used for the proof of concept for correcting the HbS mutation using prime editor.

Our study reiterates the fact that prime editing could be utilised for efficient modelling of diseases in human erythroid cell lines. However, constitutive expression over a longer period might be required to achieve bi-allelic mutation at the target site.

### Seamless correction of sickle cell mutation and reconstitution of adult haemoglobin using prime editor

**FIGURE 4:**
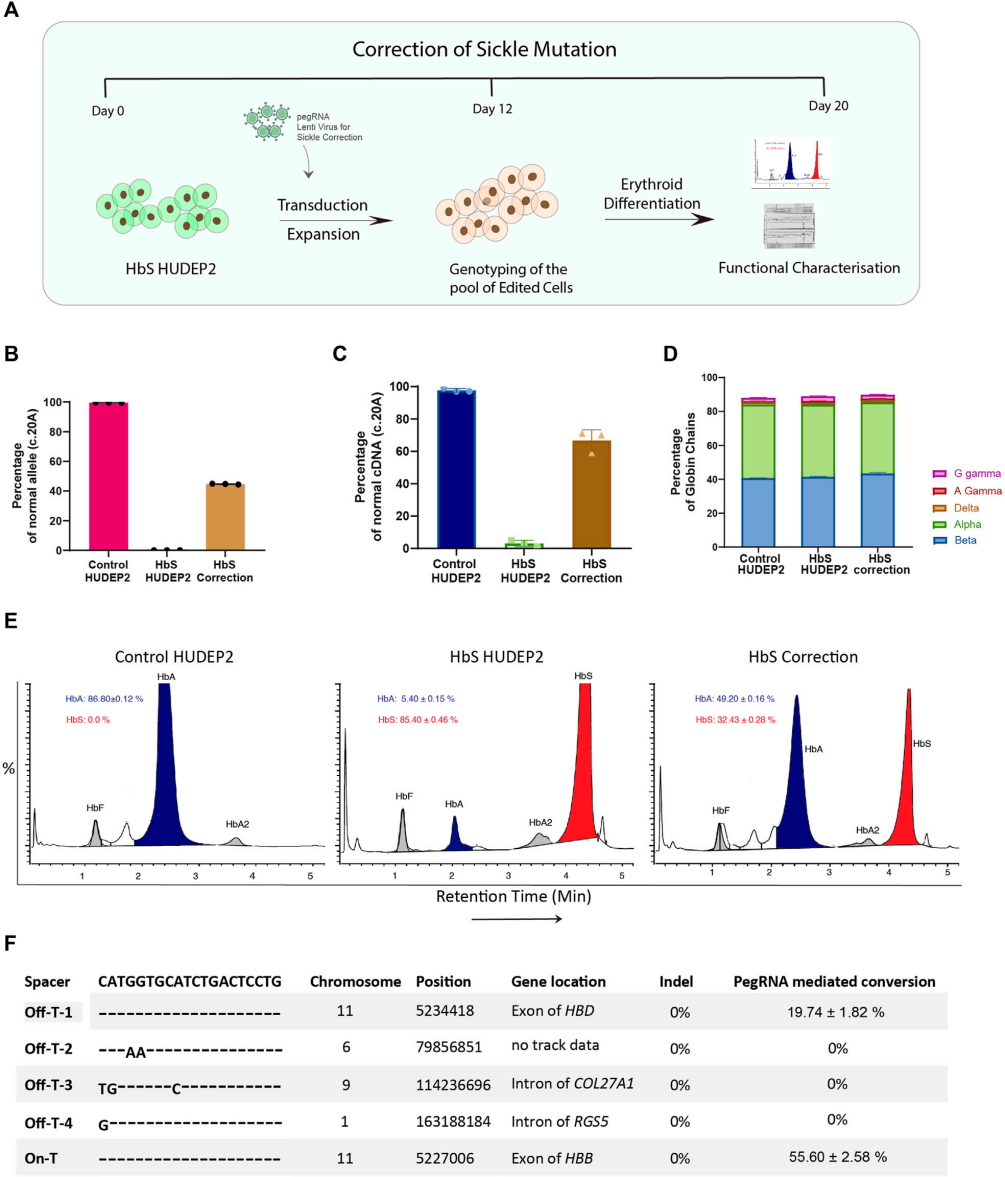
Correction of sickle cell mutation in human erythroid cells using prime editor **(A)** Schematic representation of the workflow for correction of sickle cell mutation in the HbS HUDEP2 cells. **(B)** Editing efficiency for correction of HbS mutation at Day 12 after transduction evaluated by next-generation sequencing **(C)** Correction of sickle mutation at the cDNA level evaluated by sanger sequencing **(D)** RP-HPLC analysis representing the percentage of individual globin chains upon erythroid differentiation after prime editing **(E)** Haemoglobin variant analysis by HPLC after erythroid differentiation of the edited cells. **(F)** Analysis of editing at the top 4 *in silico* predicted off-target sites using NGS compared with on-target editing for the installation of HbS mutation in the *HBB* gene. Results from three replicates are plotted as mean ± SD.

Using the created cellular model of sickle cell mutation in human erythroid cells (HbS HUDEP2 Cells), we performed the proof-of-concept evaluation for the correction of HbS mutation and restoration of adult haemoglobin production (Figure 4A). The major limitation of the generated cellular model was the constitutive expression of the initially transduced pegRNA for installation of HbS mutation even after long-term culturing owing to the lenti-viral delivery. To prevent the reconversion of the corrected alleles back to HbS mutation by the initially transduced pegRNA, we selected the pegRNA for the correction of mutation that would create a silent PAM mutation in addition to the desired T > A conversion. The pegRNA extension was 25 bp long with 12 nt Primer Binding Site (PBS) and 13 nt Reverse Transcriptase (RT) template and was similar to the one for installation of HbS mutation in all respects except at the SCA mutation and the installation of silent PAM mutation (GTG>GAA). Further, to assess the transduction efficiency of the subsequently transduced pegRNA for the correction of HbS mutation without interference from the other pegRNA, a lenti-viral vector that co-expresses RFP instead of GFP was used.

HbS HUDEP2 cells were transduced with the pegRNA for the correction of HbS mutation and the transduction efficiency was estimated using RFP expression. We used the unmodified vector as a negative control to account for any variations while performing a second round of transduction in cells with a previously integrated pegRNA. We obtained ∼100% transduction efficiency in both the experimental and vector control transduced cells, similar to what has been observed before (Supplementary Figure S4A). This data gives confidence that a second pegRNA can be delivered effectively and would express efficiently even when another pegRNA is being constitutively expressed. The transduced HbS HUDEP2 cells were maintained in culture for 12 days and the editing efficiency was evaluated by next-generation sequencing. We found that the editing efficiency of correction is comparable to the installation of HbS mutation (47%) (Figure 4B). A similar editing efficiency was observed at the cDNA level as well suggesting that the correction of HbS mutation did not affect the transcription of the *HBB* gene (Figure 4C). There was no indel production at the target site even with higher efficiency of on-target conversion (Supplementary Figure S4B). These results highlight the advantage of using the PE2 system for editing in the coding region where the indel generation will lead to disease phenotype. Further analysis with MLPA also showed the reduction of SCD mutant alleles in the edited cells (Supplementary Figure S4C).

To extensively characterise the functional effect of HbS mutation correction by prime editor, the edited cells were cultured under the erythroid differentiation conditions and analysed for the haemoglobin production. Reverse Phase HPLC analysis showed no marked difference in the production of individual globin chain levels in the edited cells compared to the control suggesting that prime editing at the target locus did not affect the translation efficiency (Figure 4D). We observed on haemoglobin variant analysis that the edited cells produce substantial amounts of wild-type adult haemoglobin (HbA: 49.20 ± 0.16% in edited cells vs. 5.40 ± 0.15% in unedited cells), with a concomitant decrease in sickle haemoglobin (HbS: 32.43 ± 0.28% in edited cells vs. 85.40 ± 0.46% in unedited cells), which corroborates with our observed on-target editing at the DNA and RNA levels (Figure 4E). There is a slight elevation in the number of HbF + cells in both the HbS mutation corrected as well as mock transduced HbS HUDEP2 cells when compared to the untransduced parental cells (Supplementary Figure S4D). As the number of HbF + levels were comparable between the two pegRNA transduced HbS HUDEP2 cells, the elevation might be due to transduction stress and might not be of importance since the levels of sickle haemoglobin and gamma globin chains detected by HPLC- analysis were not significantly altered. Since the correction is error-free and devoid of any other point mutations, we expect that the adult beta-globin produced would not sickle under hypoxic conditions.

After successfully confirming the use of prime editing system for the correction of HbS mutation and amelioration of SCA phenotype, we went on to characterise the safety profile of the pegRNA. Prime editor is considered safer than conventional gene editing tools as multiple hybridisation steps are required between spacer, PBS and RT template with their respective binding regions in DNA for successful prime editing to occur (Anzalone et al., 2019). Several studies have reported minimal off-target effects for prime editing by whole genome sequencing and targeted amplification (Kim et al., 2020; Jin et al., 2021). Nevertheless, it is important to evaluate the off-target effects for each pegRNA during preclinical validations.

To investigate the possible off-target effects, we used the unsorted edited cells that were transduced with pegRNA for installation of HbS mutation rather than the individual clones or the cell corrected of sickle mutation thereof. We reasoned that the use of unsorted edited cells would provide an unbiased view of off-target effects mediated by the pegRNA. As the spacer sequence is common between the pegRNA that is used for the creation and correction of HbS mutation, we presumed that the off-target effects would also be occurring in a similar frequency. We performed the targeted deep sequencing at the top four off-target sites predicted by the *in silico* tools. We did not observe any indels at any of the off-target sites or the on-target region. Further, we did not observe any pegRNA driven mutations at three out of four off-target sites compared to the control (Figure 4F).

The only off-target site that showed editing was the highly homologous *HBD* region almost 8 kb upstream of the *HBB* gene where the pegRNA had exact sequence homology. As expected, we noticed a considerable amount of editing at the *HBD* region (∼19%) but this frequency of conversion was significantly lower than the editing observed at the on-target *HBB* region (Figure 4F). The editing efficiency at the off target site also increased with time as has been observed at the on target site (Supplementary Figure S4E). The off-target editing will not be a concern during the correction of HbS mutation in patient samples as the WT sequence would be present at the *HBD* region. The installation of silent PAM mutation could still occur with similar efficiency at *HBD* off-target site as seen by sanger sequencing of WT cells treated with pegRNA for the correction of HbS mutation (Supplementary Figure S4F).

Previous studies with programmable nucleases have suggested that 20–30% correction of HbS mutation in the patient CD34+ HSPCs would be sufficient for the treatment of SCD. Almost fifty percent of correction achieved by prime editing predicts a higher possibility of edited cells harbouring heterozygous HbS mutation. Based on the allelic frequency obtained from the individual clones during the HbS mutation creation and considering the normal phenotype of individuals with heterozygous HbS mutation, we expect that the level of error-free conversion achieved by the prime editor would be sufficient for the therapeutic correction of SCD mutation in patients.

## Discussion

Sickle cell Disease (SCD) being a monogenic disorder and owing to the lack of other efficient curative approaches is an attractive target for gene therapy (Kato et al., 2018). Homology-directed repair and base editing have been used in the past to demonstrate the correction of SCA but both approaches have dire inherent limitations. HDR, mediated by DSB could be deleterious in the correction of SCA if the proportion of indels is high due to the risk of creating beta-thalassaemic mutations (DeWitt et al., 2016). Base editing on the other hand, cannot be used to install T>A transition mutation and has relied on the creation of Hb-G Makassar, a non-pathogenic Hb variant to overcome the SCA mutation (Newby et al., 2021). The functional efficiency of Hb-G Makassar variant has not been evaluated extensively. Prime editing overcomes both these issues by avoiding DSB and the ability to install precise T>A mutation. The proof of concept of correcting SCA using prime editing has been shown by Anzalone et al. in HEK293T cells; however, no studies have demonstrated a functional correction of HbS mutation and evaluated the safety of the use of prime editing in human erythroid cells.

Here, we sought to evaluate prime editing for the correction of HbS mutation in HUDEP2 cells using PE2 system that would install the mutation without any bystander edits or indels. So far, most of the PE studies have relied on the PE3 system, which requires a gRNA that nicks the opposite strand to achieve better editing efficiency but carries the risk of generating small insertions and deletions at the target site. Undesired edits and indels can interfere with the evaluation of the functional effects of the mutation especially in the coding regions. To improve the PE2 system in an efficient and versatile manner, we first developed a lenti-viral based approach that can edit different target sites across various human cell lines. This approach was utilised for efficiently modelling the HbS mutation in HUDEP2 cells which was further used for demonstrating the utility of prime editing in the correction of HbS mutation. Further, we comprehensively evaluated the reconstitution of functional adult-haemoglobin in the created SCA cellular model and assessed the on- and off-target safety profile of the use of prime editor in the beta-globin locus.

Delivery approaches affect prime editing efficiency to a great extent as the availability of the prime editor and pegRNA for a longer period has been shown to ensure efficient editing at the target site (Eggenschwiler et al., 2021; Wolff et al., 2021). Lenti-viral vectors offer an attractive platform for the delivery of PE as they would integrate into the genome and constitutively express the components. Previous studies have attempted to deliver pegRNA and prime editor as an all-in-one lenti-viral vector which necessitated either the development of a truncated version of PE or the delivery as split-intein based system, owing to the decrease in transduction efficiency with increased size of the vector (Anzalone et al., 2019; Gao et al., 2022). Both approaches however limited the editing efficiency considerably and a single full sized lenti-viral vector had failed to produce any editing. We aimed to overcome this problem by uncoupling PE and pegRNA delivery and developed two different constructs that can separately deliver the components. We have shown that this system is highly efficient compared to other viral and non-viral delivery approaches and is able to achieve even 100% editing at certain target sites. In addition, the dual lenti-viral based system can be used efficiently in cell lines other than HEK293T as well, expanding the targeting scope of prime editing. The potential to prepare the cell lines stably expressing PE which can then be transduced with pegRNAs enable efficient arrayed screening of multiple pegRNAs for initial optimisations. We also show that the sequential delivery of two pegRNAs does not affect the expression of either making it possible to use the lenti-viral system for studies that require dual pegRNA delivery. The developed dual lenti-viral system greatly increases the targeting scope of prime editing for preclinical studies and allows efficient comparison of pegRNAs.

Using the optimised modular lenti-viral based approach, we were able to edit difficult-to-manipulate HUDEP2 cells with editing efficiency reaching up to 75% over time using prime editor 2 system. HUDEP2 cells are important cellular model for preclinical studies involving beta-haemoglobinopathies as they can easily be differentiated into erythroid lineage and express adult haemoglobin to a quantifiable level. In addition to evaluating prime editing as a tool for the correction of mutations, this approach facilitates the use of prime editing in understanding globin biology and gene switching with its ability to introduce precise mutations with higher efficiency. Higher editing efficiency mediated by lenti-viral PE system also overcomes the need for single-cell sorting for most applications avoiding the biases associated with the clonal selection when evaluating the gene expression. We hope that the lenti-viral system can be used in other cell types as well, relevant to facilitate research in different domains.

Modelling of beta-thalassaemic mutations in HUDEP2 cells has been attempted by another research group but showed very poor efficiency (Zhang et al., 2022). We were able to install the HbS mutation with very high efficiency and obtained a high proportion of homozygous clones with SCA mutation. We also did not observe any small insertions or deletions in the nick site even when the editing efficiency had reached more than 70%. This can be attributed to the use of PE2 system, which does not generate any undesired mutations (Ferreira da Silva et al., 2022). PE2 system would be a better choice in studies involving coding regions or regions where sequence integrity needs to be preserved as the phenotypic outcomes would be an accurate reflection of the installed edit and not influenced by the by-products even to a minimal degree. The model system that we developed produced sickle haemoglobin exclusively without compromising the expression of any other globin chains. The major drawback of the developed model system is the constitutive expression of PE and pegRNA which limit its use for prime editing studies not affected by the expression of the initially transduced pegRNA. Transient delivery of prime editing components could overcome the limitation although with reduced efficiency and would require delivery as mRNA or RNP complex and the screening of a large number of clones to obtain the desired homozygous edited clones.

Correction of SCA mutation was evaluated using the developed model cell line using a pegRNA that installs a silent PAM mutation in addition to correcting the HbS mutation. The installation of the silent PAM mutation would overcome the limitation associated with the expression of the initially transduced pegRNA, which was used for the creation of HbS mutation. We demonstrated high efficiency in the correction of HbS mutation as well as validated the functional production of normal adult haemoglobin concomitant with the editing efficiency. This demonstrates the utility of prime editing for the correction of HbS mutation which could now be evaluated in SCD patient CD34+ HSPCs by optimising the delivery. During the assessment of editing at the off-target sites, although other off-target sites were not edited, we noticed a higher frequency of editing at the delta-globin gene which is highly homologous to the beta-globin. This might not have any consequence during the correction of the SCD mutation in patient’s CD34+ HSPCs as the corresponding sequence in the delta-globin would be intact. The system also provides a platform for efficient screening of pegRNAs for the installation of mutation, the best of which could be taken for further evaluation in the patient CD34+ HSPCs thereby lowering the number of primary cells required for the genetic manipulation as well as cutting down the expenditure. However, before this statement can be generalised, a comparison of prime editing efficiency at the specific locus in different cell lines and CD34+ HSPCs needs to be performed as we have observed a cell type dependent variation in editing efficiency for the same pegRNA even when the level of expression of PE and pegRNA was consistent. The resulting variation might be due to cell intrinsic factors or gene expression profiles that need a thorough evaluation to develop improved versions of PE specific to the cell type in question. In addition, we and others have noticed a consistent increase in prime editing efficiency with constitutive expression over time, hinting at the cell division dependency of prime editing which might be a bottleneck when editing primary cells (Wolff et al., 2021).

Delivery of base editors as mRNA with synthetic gRNA has been shown to edit efficiently in HSPCs. Hanqin et al. compared different delivery modalities of prime editor in Human induced pluripotent stem cells and found that PE mRNA combined with synthetic pegRNA gave the highest editing efficiency (Li et al., 2022). A similar approach would be most suitable for prime editing in HSPCs as well and the recently described modifications in the prime editor (PE max, PE4 and PE5) and pegRNA (epegRNA) could improve the overall efficiency (Chen et al., 2021; Nelson et al., 2022). The delivery of PE as Integrase Deficient Lenti Virus (IDLV) could be another viable approach for editing patient derived CD34+ HSPCs (Ortinski et al., 2017). Prime editing has been shown to generate minimal chromosomal aberrations compared to DSB mediated approaches and hence the edited cells might have better engraftment potential improving the treatment outcome.

To conclude, the current study demonstrates the development of a modular lenti-viral PE delivery system that can edit with high efficiency and precision across multiple cell lines and different genomic loci. This approach was used to precisely model and correct the HbS mutation (*HBB* E6V) without any undesirable conversion in HUDEP2 cells where functional reconstitution of beta-globin and safety of prime editing was characterised extensively for the first time. Overall, our study not only expands the application and scope of prime editing for the difficult-to-transfect cells but also provides a thorough preclinical evaluation of pegRNA for the correction of SCD. The results provide a framework for the preclinical evaluation of prime editing in CD34+ HSPCs for the correction of hematological disorders not just limited to SCD.

## Data Availability

The datasets presented in this study can be found in online repositories. The names of the repository/repositories and accession number(s) can be found below: https://www.ncbi.nlm.nih.gov/, PRJNA905550.

## References

[B1] Amendola M. Brusson M. Miccio A. (2022). CRISPRthripsis: The risk of CRISPR/Cas9-induced chromothripsis in gene therapy. Stem Cells Transl. Med. 11, 1003–1009. 10.1093/stcltm/szac064 36048170 PMC9585945

[B2] Anzalone A. V. Koblan L. W. Liu D. R. (2020). Genome editing with CRISPR–Cas nucleases, base editors, transposases and prime editors. Nat. Biotechnol. 38, 824–844. 10.1038/s41587-020-0561-9 32572269

[B3] Anzalone A. V. Randolph P. B. Davis J. R. Sousa A. A. Koblan L. W. Levy J. M. (2019). Search-and-replace genome editing without double-strand breaks or donor DNA. Nature 576, 149–157. 10.1038/s41586-019-1711-4 31634902 PMC6907074

[B4] Barbarani G. Łabedz A. Ronchi A. E. (2020). β-Hemoglobinopathies: The test bench for genome editing-based therapeutic strategies. Front. Genome Ed. 2, 571239. 10.3389/fgeed.2020.571239 34713219 PMC8525389

[B5] Brittenham G. M. Schechter A. N. Noguchi C. T. (1985). Hemoglobin S polymerization: Primary determinant of the hemolytic and clinical severity of the sickling syndromes. Blood 65, 183–189. 10.1182/blood.V65.1.183.183 3965046

[B6] Canver M. C. Smith E. C. Sher F. Pinello L. Sanjana N. E. Shalem O. (2015). BCL11A enhancer dissection by Cas9-mediated *in situ* saturating mutagenesis. Nature 527, 192–197. 10.1038/nature15521 26375006 PMC4644101

[B7] Chen P. J. Hussmann J. A. Yan J. Knipping F. Ravisankar P. Chen P.-F. (2021). Enhanced prime editing systems by manipulating cellular determinants of editing outcomes. Cell 184, 5635–5652.e29. 10.1016/j.cell.2021.09.018 34653350 PMC8584034

[B8] Clement K. Rees H. Canver M. C. Gehrke J. M. Farouni R. Hsu J. Y. (2019). CRISPResso2 provides accurate and rapid genome editing sequence analysis. Nat. Biotechnol. 37, 224–226. 10.1038/s41587-019-0032-3 30809026 PMC6533916

[B9] Conant D. Hsiau T. Rossi N. Oki J. Maures T. Waite K. (2022). Inference of CRISPR edits from sanger trace data. CRISPR J. 5, 123–130. 10.1089/crispr.2021.0113 35119294

[B46] Demirci S. Gudmundsdottir B. Li Q. Haro-Mora J. J. Nassehi T. Drysdale C. (2020). βT87Q-globin gene therapy reduces sickle hemoglobin production, allowing for ex vivo anti-sickling activity in human erythroid cells. Mol. Ther. Methods Clin. Dev. 17, 912–921. 10.1016/j.omtm.2020.04.013 32405513 PMC7210457

[B10] DeWitt M. A. Magis W. Bray N. L. Wang T. Berman J. R. Urbinati F. (2016). Selection-free genome editing of the sickle mutation in human adult hematopoietic stem/progenitor cells. Sci. Transl. Med. 8, 360ra134. 10.1126/scitranslmed.aaf9336 PMC5500303 27733558

[B11] Doman J. L. Sousa A. A. Randolph P. B. Chen P. J. Liu D. R. (2022). Designing and executing prime editing experiments in mammalian cells. Nat. Protoc. 17, 2431–2468. 10.1038/s41596-022-00724-4 35941224 PMC9799714

[B12] Eggenschwiler R. Gschwendtberger T. Felski C. Jahn C. Langer F. Sterneckert J. (2021). A selectable all-in-one CRISPR prime editing piggyBac transposon allows for highly efficient gene editing in human cell lines. Sci. Rep. 11, 22154. 10.1038/s41598-021-01689-2 34773059 PMC8589839

[B13] Esrick E. B. Lehmann L. E. Biffi A. Achebe M. Brendel C. Ciuculescu M. F. (2021). Post-transcriptional genetic silencing of BCL11A to treat sickle cell disease. N. Engl. J. Med. 384, 205–215. 10.1056/NEJMoa2029392 33283990 PMC7962145

[B14] Ferreira da Silva J. Oliveira G. P. Arasa-Verge E. A. Kagiou C. Moretton A. Timelthaler G. (2022). Prime editing efficiency and fidelity are enhanced in the absence of mismatch repair. Nat. Commun. 13, 760. 10.1038/s41467-022-28442-1 35140211 PMC8828784

[B15] Gao Z. Ravendran S. Mikkelsen N. S. Haldrup J. Cai H. Ding X. (2022). A truncated reverse transcriptase enhances prime editing by split AAV vectors. Mol. Ther. 30, 2942–2951. 10.1016/j.ymthe.2022.07.001 35808824 PMC9481986

[B16] Jin S. Lin Q. Luo Y. Zhu Z. Liu G. Li Y. (2021). Genome-wide specificity of prime editors in plants. Nat. Biotechnol. 39, 1292–1299. 10.1038/s41587-021-00891-x 33859403

[B17] Kanter J. Walters M. C. Krishnamurti L. Mapara M. Y. Kwiatkowski J. L. Rifkin-Zenenberg S. (2022). Biologic and clinical efficacy of LentiGlobin for sickle cell disease. N. Engl. J. Med. 386, 617–628. 10.1056/NEJMoa2117175 34898139

[B18] Kato G. J. Piel F. B. Reid C. D. Gaston M. H. Ohene-Frempong K. Krishnamurti L. (2018). Sickle cell disease. Nat. Rev. Dis. Prim. 4, 18010. 10.1038/nrdp.2018.10 29542687

[B19] Kavanagh P. L. Fasipe T. A. Wun T. (2022). Sickle cell disease: A review. JAMA 328, 57–68. 10.1001/jama.2022.10233 35788790

[B20] Kim D. Y. Moon S. B. Ko J.-H. Kim Y.-S. Kim D. (2020). Unbiased investigation of specificities of prime editing systems in human cells. Nucleic Acids Res. 48, 10576–10589. 10.1093/nar/gkaa764 32941652 PMC7544197

[B21] Kluesner M. G. Nedveck D. A. Lahr W. S. Garbe J. R. Abrahante J. E. Webber B. R. (2018). EditR: A method to quantify base editing from sanger sequencing. CRISPR J. 1, 239–250. 10.1089/crispr.2018.0014 31021262 PMC6694769

[B22] Kurita R. Suda N. Sudo K. Miharada K. Hiroyama T. Miyoshi H. (2013). Establishment of immortalized human erythroid progenitor cell lines able to produce enucleated red blood cells. PLOS ONE 8, e59890. 10.1371/journal.pone.0059890 23533656 PMC3606290

[B23] Leibowitz M. L. Papathanasiou S. Doerfler P. A. Blaine L. J. Sun L. Yao Y. (2021). Chromothripsis as an on-target consequence of CRISPR-Cas9 genome editing. Nat. Genet. 53, 895–905. 10.1038/s41588-021-00838-7 33846636 PMC8192433

[B24] Li H. Busquets O. Verma Y. Syed K. M. Kutnowski N. Pangilinan G. R. (2022). Highly efficient generation of isogenic pluripotent stem cell models using prime editing. eLife 11, e79208. 10.7554/eLife.79208 36069759 PMC9584603

[B25] Lu C. Kuang J. Shao T. Xie S. Li M. Zhu L. (2022). Prime editing: An all-rounder for genome editing. Int. J. Mol. Sci. 23, 9862. 10.3390/ijms23179862 36077252 PMC9456398

[B26] Magrin E. Miccio A. Cavazzana M. (2019). Lentiviral and genome-editing strategies for the treatment of β-hemoglobinopathies. Blood 134, 1203–1213. 10.1182/blood.2019000949 31467062

[B27] Morgan R. A. Gray D. Lomova A. Kohn D. B. (2017). Hematopoietic stem cell gene therapy: Progress and lessons learned. Cell Stem Cell 21, 574–590. 10.1016/j.stem.2017.10.010 29100011 PMC6039108

[B28] Nelson J. W. Randolph P. B. Shen S. P. Everette K. A. Chen P. J. Anzalone A. V. (2022). Engineered pegRNAs improve prime editing efficiency. Nat. Biotechnol. 40, 402–410. 10.1038/s41587-021-01039-7 34608327 PMC8930418

[B29] Newby G. A. Yen J. S. Woodard K. J. Mayuranathan T. Lazzarotto C. R. Li Y. (2021). Base editing of haematopoietic stem cells rescues sickle cell disease in mice. Nature 595, 295–302. 10.1038/s41586-021-03609-w 34079130 PMC8266759

[B30] Ortinski P. I. O’Donovan B. Dong X. Kantor B. (2017). Integrase-deficient lentiviral vector as an all-in-one platform for highly efficient CRISPR/Cas9-Mediated gene editing. Mol. Ther. Methods Clin. Dev. 5, 153–164. 10.1016/j.omtm.2017.04.002 28497073 PMC5424571

[B31] Papathanasiou S. Markoulaki S. Blaine L. J. Leibowitz M. L. Zhang C.-Z. Jaenisch R. (2021). Whole chromosome loss and genomic instability in mouse embryos after CRISPR-Cas9 genome editing. Nat. Commun. 12, 5855. 10.1038/s41467-021-26097-y 34615869 PMC8494802

[B32] Pawliuk R. Westerman K. A. Fabry M. E. Payen E. Tighe R. Bouhassira E. E. (2001). Correction of sickle cell disease in transgenic mouse models by gene therapy. Science 294, 2368–2371. 10.1126/science.1065806 11743206

[B33] Petri K. Zhang W. Ma J. Schmidts A. Lee H. Horng J. E. (2021). CRISPR prime editing with ribonucleoprotein complexes in zebrafish and primary human cells. Nat. Biotechnol. 40, 189–193. 10.1038/s41587-021-00901-y 33927418 PMC8553808

[B34] Platt O. S. Orkin S. H. Dover G. Beardsley G. P. Miller B. Nathan D. G. (1984). Hydroxyurea enhances fetal hemoglobin production in sickle cell anemia. J. Clin. Invest. 74, 652–656. 10.1172/JCI111464 6205021 PMC370519

[B35] Prasad K. George A. Ravi N. S. Mohankumar K. M. (2021). CRISPR/Cas based gene editing: Marking a new era in medical science. Mol. Biol. Rep. 48, 4879–4895. 10.1007/s11033-021-06479-7 34143395 PMC8212587

[B36] Psatha N. Reik A. Phelps S. Zhou Y. Dalas D. Yannaki E. (2018). Disruption of the BCL11A erythroid enhancer reactivates fetal hemoglobin in erythroid cells of patients with β-thalassemia major. Mol. Ther. Methods Clin. Dev. 10, 313–326. 10.1016/j.omtm.2018.08.003 30182035 PMC6120587

[B37] Ravi N. S. Wienert B. Wyman S. K. Bell H. W. George A. Mahalingam G. (2022). Identification of novel HPFH-like mutations by CRISPR base editing that elevate the expression of fetal hemoglobin. eLife 11, e65421. 10.7554/eLife.65421 35147495 PMC8865852

[B38] Ribeil J.-A. Hacein-Bey-Abina S. Payen E. Magnani A. Semeraro M. Magrin E. (2017). Gene therapy in a patient with sickle cell disease. N. Engl. J. Med. 376, 848–855. 10.1056/NEJMoa1609677 28249145

[B39] Salinas Cisneros G. Thein S. L. (2020). Recent advances in the treatment of sickle cell disease. Front. Physiol. 11, 435. 10.3389/fphys.2020.00435 32508672 PMC7252227

[B40] Schene I. F. Joore I. P. Oka R. Mokry M. van Vugt A. H. M. van Boxtel R. (2020). Prime editing for functional repair in patient-derived disease models. Nat. Commun. 11, 5352. 10.1038/s41467-020-19136-7 33097693 PMC7584657

[B41] Sheth S. Licursi M. Bhatia M. (2013). Sickle cell disease: Time for a closer look at treatment options? Br. J. Haematol. 162, 455–464. 10.1111/bjh.12413 23772687

[B42] Wolff J. H. Haldrup J. Thomsen E. A. Andersen S. Mikkelsen J. G. (2021). piggyPrime: High-Efficacy prime editing in human cells using piggyBac-based DNA transposition. Front. Genome Ed. 3, 786893. 10.3389/fgeed.2021.786893 34870275 PMC8633390

[B43] Zafra M. P. Schatoff E. M. Katti A. Foronda M. Breinig M. Schweitzer A. Y. (2018). Optimized base editors enable efficient editing in cells, organoids and mice. Nat. Biotechnol. 36, 888–893. 10.1038/nbt.4194 29969439 PMC6130889

[B44] Zeng J. Wu Y. Ren C. Bonanno J. Shen A. H. Shea D. (2020). Therapeutic base editing of human hematopoietic stem cells. Nat. Med. 26, 535–541. 10.1038/s41591-020-0790-y 32284612 PMC7869435

[B45] Zhang H. Zhou Q. Chen H. Lu D. (2022). Prime editor 3 mediated beta-thalassemia mutations of the HBB gene in human erythroid progenitor cells. Int. J. Mol. Sci. 23, 5002. 10.3390/ijms23095002 35563395 PMC9099916

