# Supplementary material for: Efficient and error-free correction of sickle mutation in human erythroid cells using prime editor-2
Source: Front Genome Ed. 2022 Dec 20;4:1085111. doi: 10.3389/fgeed.2022.1085111 (PMC9808041; doi:10.3389/fgeed.2022.1085111)
Supplement: Supplementary file 1 [file Table1.DOCX]

| Sl No | Name | Spacer (5’ TO 3’) | Extension | Source |
| --- | --- | --- | --- | --- |
| 1 | HEK_CTT | caccGGCCCAGACTGAGCACGTGAgtttt | gtgcTCTGCCATCAAAGCGTGCTCAGTCTG | Anzalone et al; 2019 |
| 2 | RNF_C>G | caccGTCATCTTAGTCATTACCTGgtttt | gtgcAACGAACACCTCACGTAATGACTAAGATG | Anzalone et al; 2019 |
| 3 | HEXA_TATC | caccGTACCTGAACCGTATATCCTAgtttt | gtgcAGTCAGGGCCATAGGATAGATATACGGTTC | Anzalone et al; 2019 |
| 4 | HEK_HBB_INS | caccGCATGGTGCACCTGACTCCTGgtttt | gtgcAGACTTCTCCACAGGAGTCAGGTGCAC | Anzalone et al; 2019 |
| 5 | HUDEP_HBB_INS | caccGCATGGTGCATCTGACTCCTGgtttt | gtgcAGACTTCTCCACAGGAGTCAGATGCAC | This study |
| 6 | HUDEP_HBB_COR | caccGCATGGTGCATCTGACTCCTGgtttt | gtgcAGACTTCTCTTCAGGAGTCAGATGCAC | This study |
|  |  |  |  |  |

|  |  |  |
| --- | --- | --- |

**PegRNAs used in this study**

**Sanger Sequencing Primers used in this study**

| Sl No | Name | Forward Primer | Reverse Primer | Source |
| --- | --- | --- | --- | --- |
| 1 | HEK3_San | ATGTGGGCTGCCTAGAAAGG | CCCAGCCAAACTTGTCAACC | Anzalone et al; 2019 |
| 2 | RNF_San | ACGTCTCATATGCCCCTTGG | GTAGGAATTTTGGTGGGACA | Anzalone et al; 2019 |
| 3 | HEXA_San | CATACAGGTGTGGCGAGAGG | CCAGCCTCCTTTGGTTAGCA | Anzalone et al; 2019 |
| 4 | HBB_San | GTCATCACTTAGACCTCACC | CTGTACCCTGTTACTTATCC | This study |
| 5 | HBB_cDNA_San | ACATTTGCTTCTGACACA | TCTGGATTCTGCCTAATAAA | This study |
| 6 | Colony PCR | GAGGGCCTATTTCCCATGAT | TGGATCTCTGCTGTCCCTGT | Ravi et al; 2022 |

**NGS primers used in this study**

| Sl No | Name | Forward Primer | Reverse Primer |
| --- | --- | --- | --- |
| 1 | HBB_NGS_1 | CTATTGGTCTCCTTAAACCTGTCTTGTAACCT | AGGAGCAGGGAGGGCAGGA |
| 2 | HBB_NGS_2 | GCTTCTGACACAACTGTGTT | TCTTCTCTGTCTCCACATGC |
| 3 | HBD_NGS(OT_1) | CCATTTGCCTCCTTGAGCCTCT | TCACTGGAGCAGGGAGGACA |
| 4 | OT_2 | CATATGAACCTAATTACCTCCCAAAGGTCTC | CTCCTGCTGTAAAACTTGCCTTGG |
| 5 | OT_3 | CCTGCCTGACACCCCTATAG | CTCTTCCTCTTCCCTCCCTC |
| 6 | OT_4 | CGGAAAATACGGCTTGAGGAGAG | CTGAGGTCTAACCCAGCACTTTG |

**RT PCR primers used in this study**

| Sl No | Name | Forward Primer | Reverse Primer | Remarks |
| --- | --- | --- | --- | --- |
| 1 | Cas9_RT | CCGAAGAGGTCGTGAAGAAG | GCCTTATCCAGTTCGCTCAG | Fig 2.C/D |
| 2 | FIX_RT | ACATTGCCCTTCTGGAACTG | TCCCCAGCCACTTACATAGC | Fig 2.C |
| 3 | GAPDH_RT | CTGCACCACCAACTGCTTAG | GTCTTCTGGGTGGCAGTGAT | Fig 2.D |
|  |  |  |  |  |

**Primers used for cloning the Lenti viral vectors**

| Sl No | Name | Forward Primer | Reverse Primer | Remarks |
| --- | --- | --- | --- | --- |
| 1 | PE2_Ins_LV | GCCAGAACACAGGTGTCGTGACGCGTCTAGAGCCACCATGAAACGGACAGCCGACGGAAGCG | GTGGCGCCGCTGCCGCTAGCATGCATGACTTTCCTCTTCTTCTTGG | For Gibson Assembly |
| 2 | Peg_Acc_LV | TAAAATGGACTATCATATGCTTACCGTAAC | CTAGAATTCAAAAAAAGAGACGGTCACGTC | For Gibson Assembly |
|  |  |  |  |  |
