# Supplementary material for: Efficient and error-free correction of sickle mutation in human erythroid cells using prime editor-2
Source: Front Genome Ed. 2022 Dec 20;4:1085111. doi: 10.3389/fgeed.2022.1085111 (PMC9808041; doi:10.3389/fgeed.2022.1085111)
Supplement: Supplementary file 2 [file DataSheet1.pdf]

Supplementary Figure 1

A)

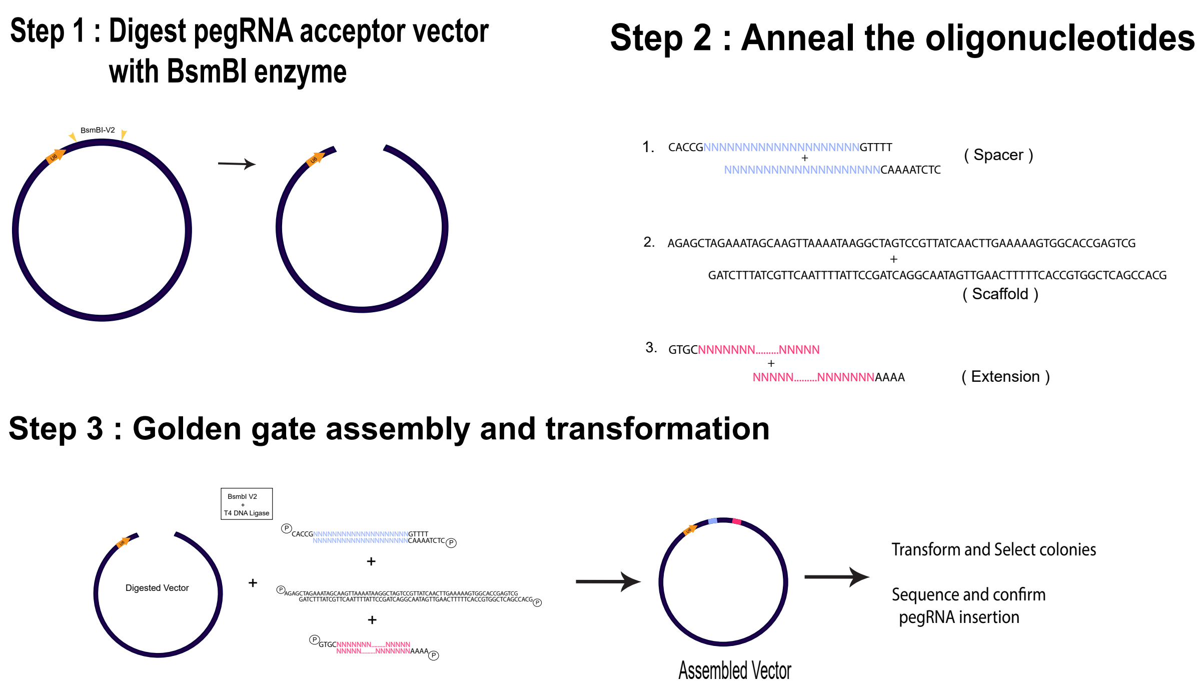

B)

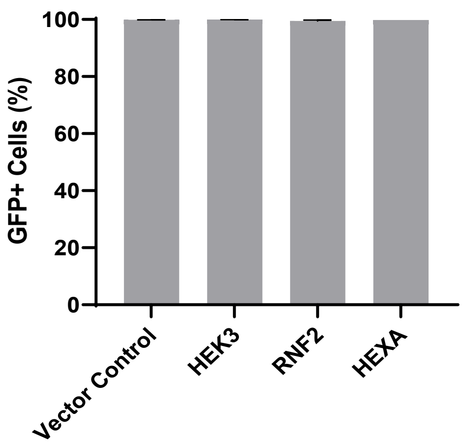

Supplementary Figure 1: Highly efficienct lentiviral delivery of pegRNAs in HEK293T cells. A) Schematic representation of the worklfow for cloning of pegRNA into the lentiviral acceptor vector. **B)** Transduction efficiency of different pegRNAs in HEK293T cells evaluated by GFP expression using FACS. Results from three replicates are plotted as mean± SD;

Supplementary Figure 2

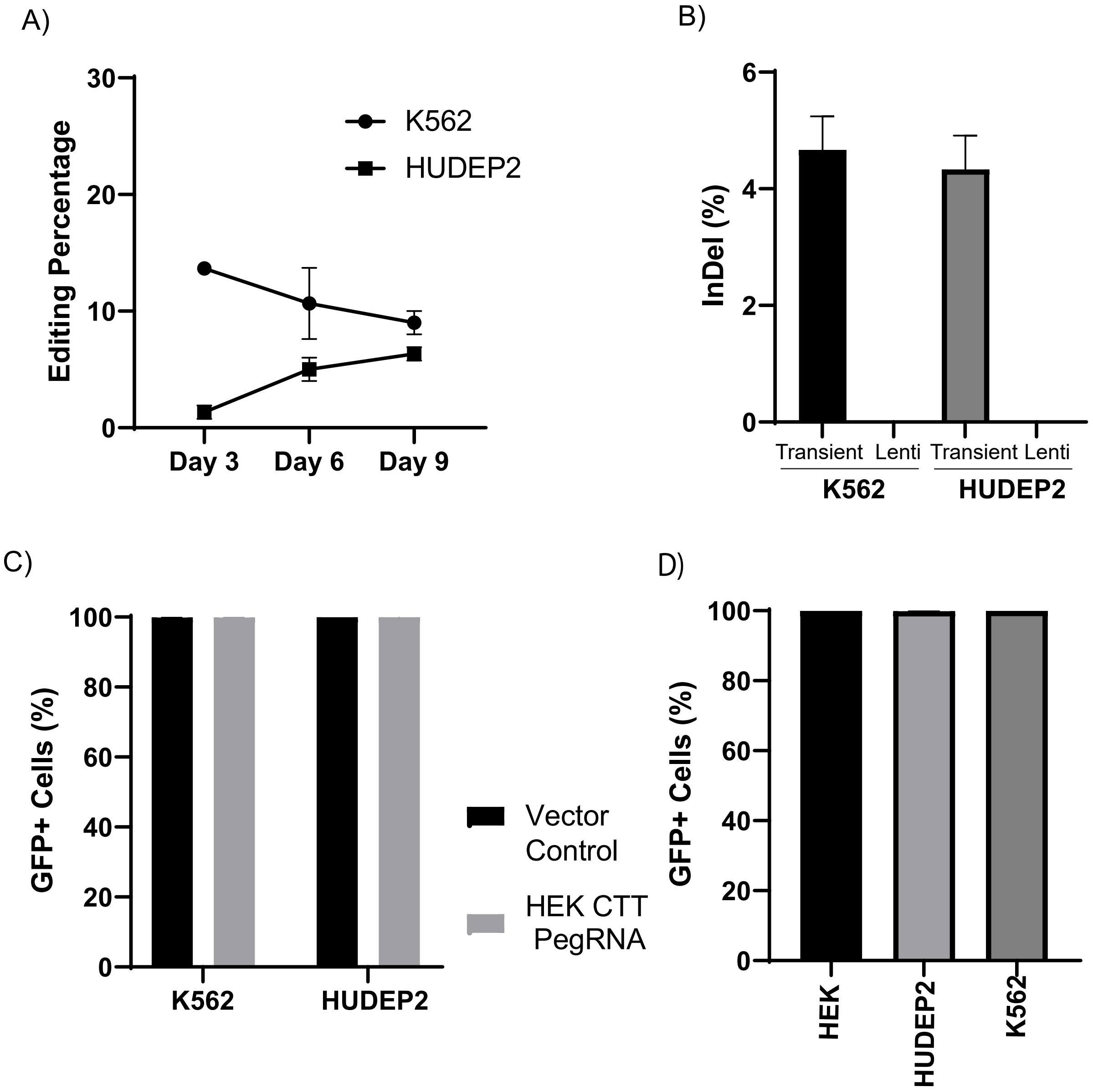

Supplementary Figure 2 : Evaluation of delivery efficiency and indel in erythroid cell lines after prime editing A) Editing efficiency overtime in K562 and HUDEP2 cells for CTT insertion in HEK3 locus by transient delivery of pegRNA. **B**)Comparison of Indels produced in HEK3 locus as a by-product of CTT insertion via transient vs lenti viral delivery. **C)** Transduction efficiency of pegRNA for CTT insertion in HEK3 locus measured by GFP expression using flow cytometry **D)** Transducion efficieny of pegRNA for HBB E6V installation in the beta globin gene. Results from three replicates are plotted as mean± SD;

Supplementary Figure 3

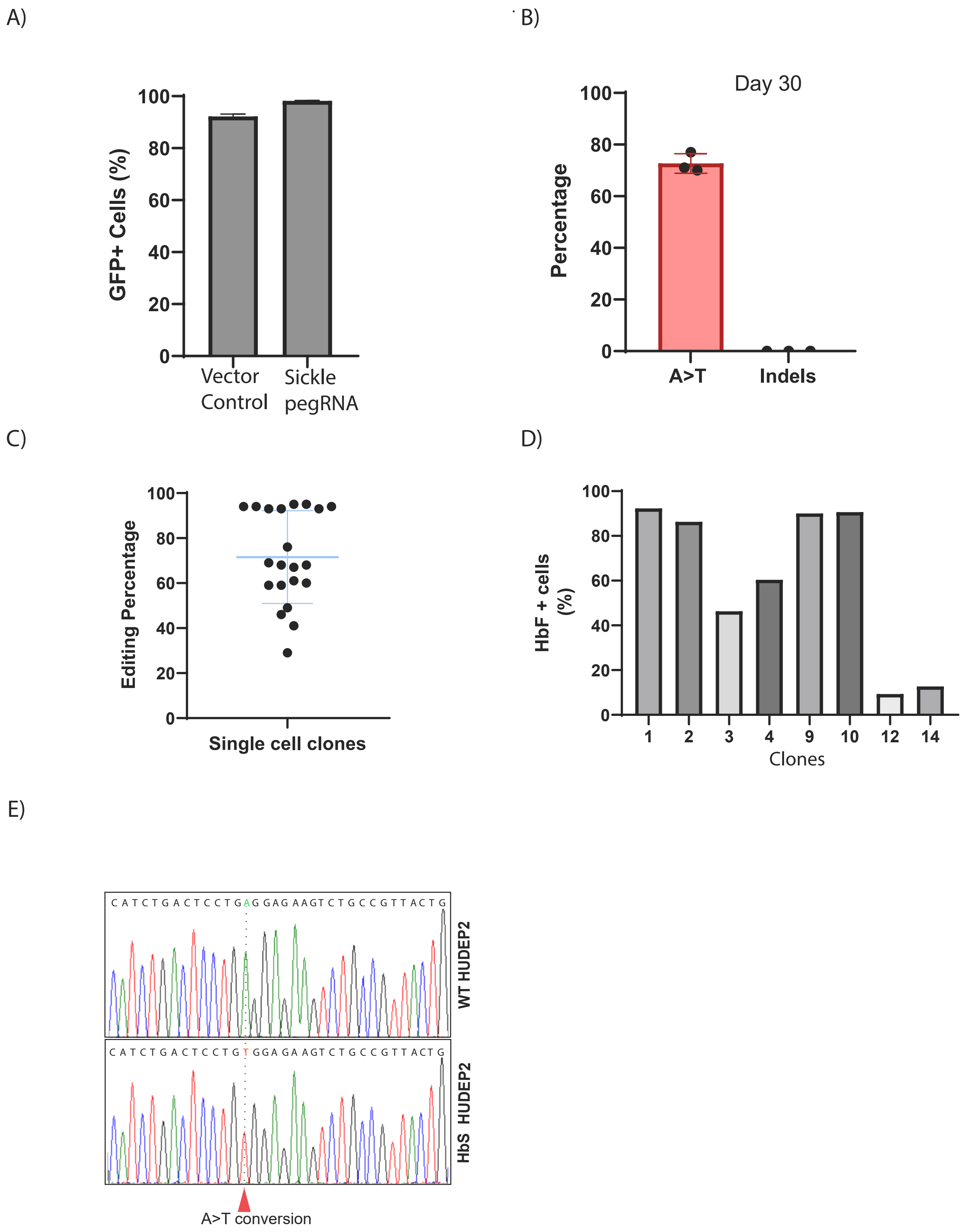

Supplementary Figure 3: Creation of sickle mutant cell line A) Transduction efficiency of the pegRNAs assessed by GFP expression via flow cytometry **B)** Comparison of the percentage of pure prime edits vs Indels for creation of sickle mutation 30 days after transduction. **C)** Editing percentage of individual clonal population after single cell sorting. **D)** Percentage of HbF positive cells in the 8 homozygous sickle clones assessed by Flow cytometry. **E)** Representative Sanger sequencing chromatogram for homozygous edited clone showing complete conversion at the target site. Results from three replicates are plotted as mean± SD;

Supplementary Figure 4

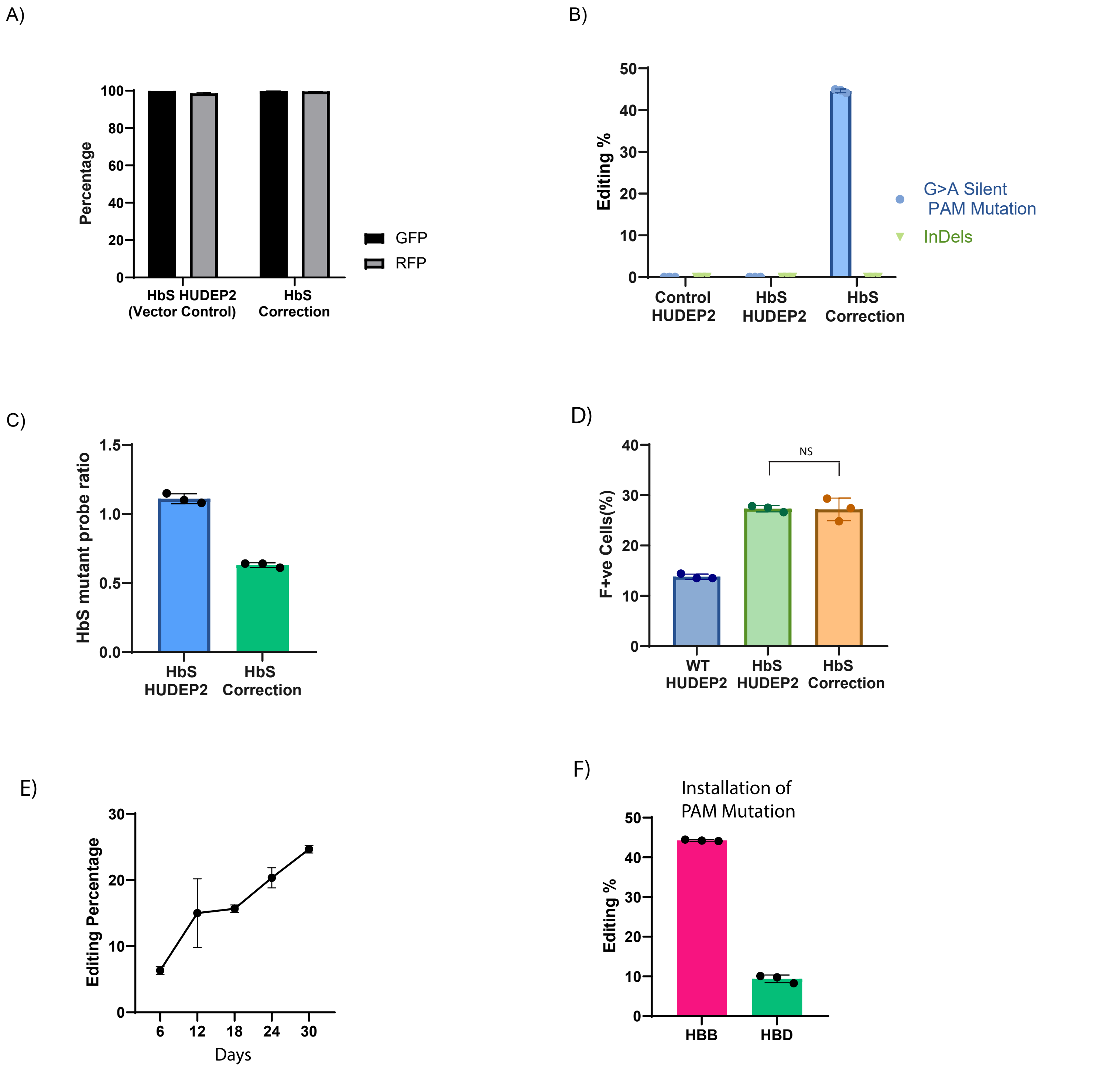

Supplementary Figure 4: Sickle mutation correction in erythroid cell line A) Evaluation of pegRNA transduction efficiency by RFP expression using FACS. **B)** Small insertions/deletions (InDels) and installation of silent PAM mutation during correction of HbS mutation using prime editing evaluated by NGS **C)** Ratio of amplification of probe specific to HbS mutation before and after correction of HbS mutation analysed by MLPA **D)** Analysis of the percentage of F+ cells by FACS after intracellular staining. **E)** Editing at the HBD off- target site over a period of 30 days. **F)** Efficiency of installation of silent PAM mutation in HBB and HBD genes independent of sickle mutation correction.Results from three replicates are plotted as mean± SD; asterisks indicate the level of statistical significance; Student t-test was used for statistical analysis (NS: non-significant)
